# Impacts of Edaphic Factors on Communities of Ammonia-Oxidizing Archaea, Ammonia-Oxidizing Bacteria and Nitrification in Tropical Soils

**DOI:** 10.1371/journal.pone.0089568

**Published:** 2014-02-28

**Authors:** Vidya de Gannes, Gaius Eudoxie, William J. Hickey

**Affiliations:** 1 Dept. Food Production, Faculty of Food and Agriculture, University of the West Indies, St. Augustine Campus, Republic of Trinidad and Tobago; 2 O.N. Allen Laboratory for Soil Microbiology, Dept. Soil Science, University of Wisconsin-Madison, Madison, Wisconsin, United States of America; University of Glasgow, United Kingdom

## Abstract

Nitrification is a key process in soil nitrogen (N) dynamics, but relatively little is known about it in tropical soils. In this study, we examined soils from Trinidad to determine the edaphic drivers affecting nitrification levels and community structure of ammonia-oxidizing bacteria (AOB) and ammonia-oxidizing archaea (AOA) in non-managed soils. The soils were naturally vegetated, ranged in texture from sands to clays and spanned pH 4 to 8. The AOA were detected by qPCR in all soils (*ca.* 10^5^ to 10^6^ copies archaeal *amoA* g^−1^ soil), but AOB levels were low and bacterial *amoA* was infrequently detected. AOA abundance showed a significant negative correlation (*p*<0.001) with levels of soil organic carbon, clay and ammonium, but was not correlated to pH. Structures of AOA and AOB communities, as determined by *amoA* terminal restriction fragment (TRF) analysis, differed significantly between soils (*p*<0.001). Variation in AOA TRF profiles was best explained by ammonium-N and either Kjeldahl N or total N (*p*<0.001) while variation in AOB TRF profiles was best explained by phosphorus, bulk density and iron (*p*<0.01). In clone libraries, phylotypes of archaeal *amoA* (predominantly *Nitrososphaera*) and bacterial *amoA* (predominanatly *Nitrosospira*) differed between soils, but variation was not correlated with pH. Nitrification potential was positively correlated with clay content and pH (*p*<0.001), but not to AOA or AOB abundance or community structure. Collectively, the study showed that AOA and AOB communities were affected by differing sets of edaphic factors, notably that soil N characteristics were significant for AOA, but not AOB, and that pH was not a major driver for either community. Thus, the effect of pH on nitrification appeared to mainly reflect impacts on AOA or AOB activity, rather than selection for AOA or AOB phylotypes differing in nitrifying capacity.

## Introduction

Nitrification is a key process in the nitrogen (N) cycle, as transformation of ammonium-N to nitrate-N can cause nitrate contamination of groundwater, and greenhouse gas production (*i.e.*, N_2_O) directly and indirectly *via* denitrification. Thus, predicting the potential for N contamination of the environment necessitates a thorough understanding of the environmental factors affecting the prokaryotes mediating nitrification, a group that now includes ammonia-oxidizing archaea (AOA). The AOA are ubiquitous constituents of marine and terrestrial environments [1], [2], [3], [4], and their discovery has changed the paradigm of aerobic nitrification mediated solely by ammonia-oxidizing bacteria (AOB). As such, for soils, there's now much interest in discerning the role AOA may play in nitrification relative to AOB, and predicting nitrification now requires an understanding of the environmental characteristics that may drive the structure and activity of both AOA and AOB communities (niche differentiation).

In soils, drivers of niche differentiation of AOA *vs.* AOB has centered largely on pH and, to a lesser extent, levels and forms of nitrogen. The former has been implicated to differentially shape AOA *vs.* AOB communities in strongly acidic soils, where AOA abundance increases, or remains unchanged, with decreasing pH, while that of AOB decreases [5], [6], [7], [8]. However, in circum-neutral to alkaline soils, correlations of AOA or AOB abundance to pH have not been consistent [9], [10]. Phylogenetic analyses have also implicated pH as a niche-shaping factor as some AOA phylotypes have been correlated with pH [11]. But, the strength of that correlation is uncertain in part because as the data set is skewed toward acidic environments, and it's often uncertain whether the correlations are attributed to pH alone and or other edaphic factors [5].

Potential differences between AOA and AOB in N relations have been evaluated by field and microcosm studies, in which large inputs of ammonium stimulated AOB, but not AOA [9]. In contrast, growth of AOA, but not AOB, was stimulated by addition of an organic N source [12]. These differences have been interpreted as reflecting a preference of AOA for low ammonium environments, which would be consistent with some physiological analyses of the few available AOA cultures [13], [14]. But, field data establishing correlations of AOA to natural levels of ammonium or any other form of soil N are limited.

Soil is a highly complex microbial habitat and it's likely that AOA and AOB communities are affected by multiple edaphic factors [7], [15], [16]. Thus, studies have recently begun to focus on identification of multiple drivers of AOA and AOB community structure and function [17]. However, to date these studies have primarily focused on comparison of land use including soils with N inputs derived from management practices and/or varying in vegetation history [18], [19], [20], [21], [22]. Also, prior studies have spanned spatial scales sufficiently large as to include climatic variability as well [17], [23].There has been relatively little focus specifically on effects of edaphic properties on AOA and AOB communities in soils with a common type of natural vegetation, and within a common climatic region. Furthermore, database surveys have revealed a strong biogeographical component to the distribution of AOA and AOB phylotypes [24], [25]. But, the environmental characteristics affecting the biogeography of AOA and AOB are ill-defined, in part because the majority of studies on soil AOA and AOB communities have focused on soils from temperate regions, and relatively little on other biomes, such as the tropics. Studies of nitrifier communities in tropical soils could add much needed information on the biogeography of AOA and AOB. Moreover, a deeper understanding of nitrification in soils of the tropics is important as N cycling in these soils impacts food production for a large part of the world, and the dynamics of global climate change.

Thus, goals of the present study were to: 1) elucidate the structure of AOA and AOB communities in tropical soils from Trinidad, 2) identify edaphic drivers of variation in their composition and 3) determine the relation between nitrification levels, edaphic characteristics and AOA/AOB communities. The soils ranged from pH 4.8 to 8.2, encompassed a range of characteristics and were all sampled from non-managed locations under natural vegetation (primarily grasses) so that anthropogenic impacts were minimized. Soils were characterized for nitrification potential and a wide range of physico-chemical characteristics, while AOA and AOB communities were evaluated for abundance *via* quantitative PCR of bacterial *amoA* and archaeal *amoA*, and community structure assessed by *amoA* terminal restriction fragment analyses. Our hypotheses were: 1) soil pH would be the major factor affecting nitrifier communities, with acidic environments selecting for greater abundance of AOA and AOA phylotypes unique to low pH soils, and 2) nitrification levels would be correlated to pH as well as the abundance of AOA in acidic soils, and to abundance of AOB in alkaline soils.

## Materials and Methods

### Soil sampling, physical and chemical properties

**Figure 1:**
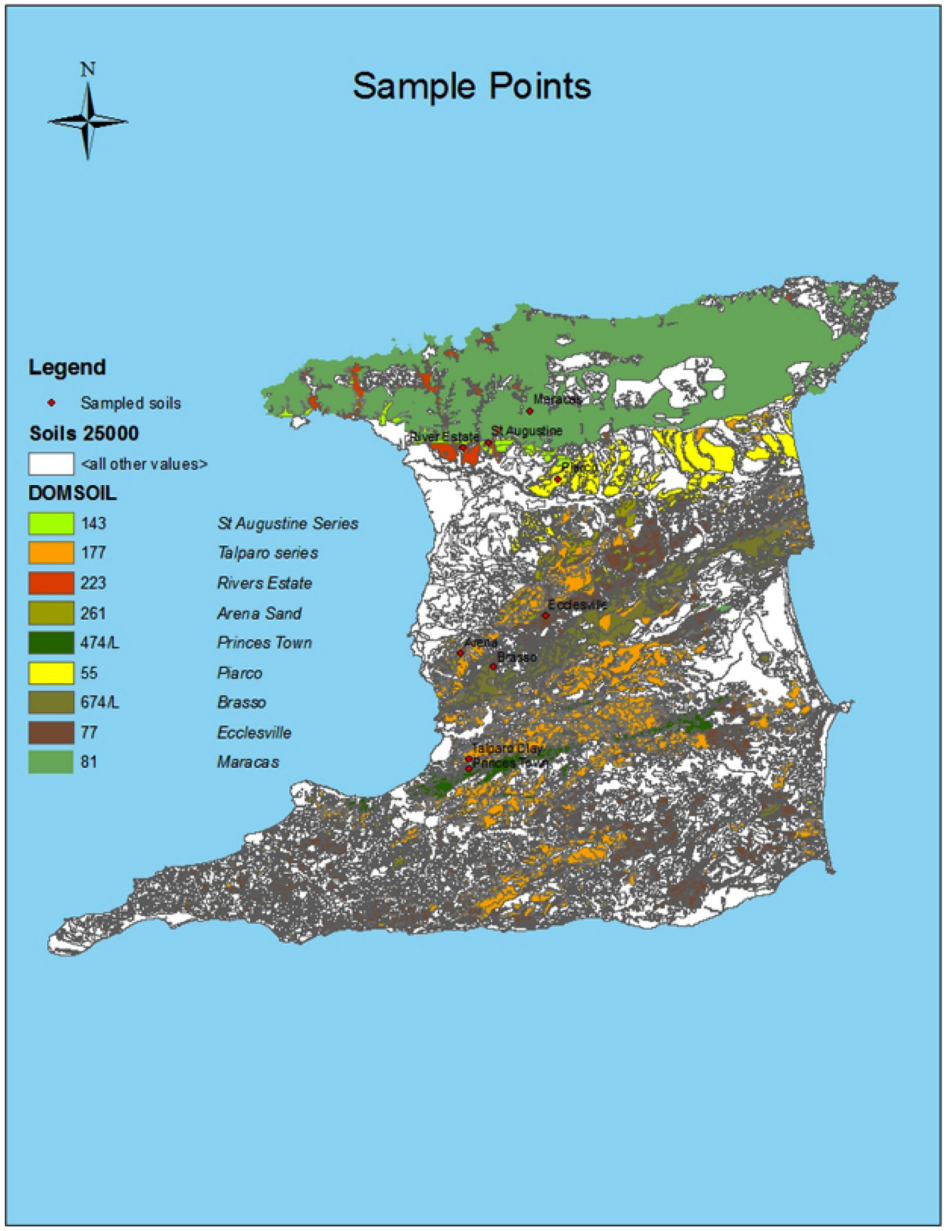
Map of Trinidad showing distribution of the soil series analyzed and the sampling locations.

Soils were collected from nine locations in Trinidad (Fig. 1, Table S1 in File S1). All soils were obtained from public land, for which no permissions were required for sampling, and no impacts on endangered species. At each location, three core samples (2.5 cm diameter×10 cm length) were obtained and the top 0–10 cm were removed for analysis. Samples were composited, mixed, sieved (4.75 mm mesh size) and subsamples (20 g each) were taken for moisture content determination by weight loss after drying (105°C, 24 h) and for physical and chemical analyses (three subsamples per soil, 50 g each). Soil samples used for the nitrification potential assay (see below) were used immediately after field sampling. Soil pH was determined by the slurry method (1∶5, w∶v; sample∶distilled deionized water) measured with an Eijkelkamp pH/mV/EC/Salinity/T/02m (Agrisearch Equipment ZG Giesbeek, the Netherlands). Total carbon (TC), organic carbon (OC) and inorganic carbon were determined by dry combustion using a LECO CNS-2000 analyzer. Total N (TN) was determined by combustion and by the Kjeldahl digestion method (TKN). Nitrate-N was measured in soil water extracts colorimetrically after reaction with phenoldisulphonic acid, and ammonium-N was determined by flow injection analysis. Major and minor trace elements were determined by inductively-coupled mass spectrometry. Cation exchange capacity (CEC) was determined by ammonium acetate extraction preceded by cation quantification *via* atomic absorption spectrometry.

### Nitrification potential

Nitrification potential (NP) was determined using the shaken slurry procedure [26]. For each sample, 5 g soil was added to 100 mL assay solution (1 mM phosphate buffer adjusted to match pH of each soil, 800 µM ammonium) and then the flasks were loosely covered and incubated with mixing (150 rpm on a bench top orbital shaker) for 24 h, at 29°C, a temperature which approximated that of the soil. The experiment was conducted with 3 replicates per soil in a 9×3 randomized design. During incubation, a sterile pipet was used to remove 10 mL aliquots at 15 min, 2 h, 8 h and 24 h intervals. These samples were centrifuged (10,000× *g*, 10 min) and the supernatant were analyzed for nitrite and nitrate [27]. Nitrite and nitrate concentrations in the 15 min interval were used as baseline measurements for which nitrite and nitrate concentrations in all later time points were corrected.

### DNA extraction, qPCR and *amoA* terminal restriction fragment analyses

The Power Soil DNA Isolation kit (MO BIO Laboratories Inc., West Carlsbad, CA) was used for DNA extraction and post-extraction clean-up was done with the Power Clean DNA Clean up kit (MO BIO Laboratories), with both procedures following the manufacturer's instructions. For each soil, triplicate 1 g soil sample extractions were done in batches of 0.25 g, giving 12 separate DNA extracts. These extracts were then pooled to give three samples (each the equivalent of one gram soil) and DNA concentrations were quantified fluorometrically by using a Quibit fluorometer (Invitrogen, Carlsbad, CA).

Each of the three replicate DNA extracts was analyzed by qPCR as described previously [1] using iQ SYBRgreen Supermix (BioRad Laboratories Inc., Hercules CA) and a MyiQ Real-Time qPCR detection system (Bio-Rad). Primer pairs used for qPCR of archaeal *amoA* were amoA_AF, amoA_AR [1], and bacterial *amoA* was amplified with primer pairs amoA_BF, amoA_BR [1]. The qPCR reactions for *amoA* quantification were done following the protocol of De Gannes et al. [1]. The qPCR analyses of archaeal 16S rRNA genes utilized primers 967F and 1060R [28] while qPCR of bacterial 16S rRNA genes was done with primers 338F and 518R [28]. Methods for qPCR of 16S rRNA genes followed those of Karlsson et al. [28]. All qPCR assays included a melting-curve protocol for analysis of primer specificity. Standard curves for qPCR were done with cloned segments of the gene of interest, with efficiencies of 91% to 100%, and r^2^ values of 98% to 99.6%. Spiking experiments in which a dilution series of standard template was prepared in the sample matrix were done to verify the absence of PCR inhibitors.

Terminal restriction fragment (TRF) analyses utilized 6FAM-labelled forward primers for archael *amoA* and bacterial *amoA* and Phusion High Fidelity Master Mix with HF buffer (New England Biolabs, Ipswich, MA). Bacterial *amoA* was amplified using the primers and assay conditions of Horz et al. [29] while PCR of archaeal *amoA* was done with the primers and conditions of Francis et al. [30]. Triplicate PCR were done for each soil by using an Eppendorf MasterCycler (Eppendorf, Hauppauge, NY), the PCR products were pooled, examined by electrophoresis, purified (QIAquick PCR Purification Kit; QIAGEN, Germantown, MD) and DNA concentrations determined fluorometrically as described above. Aliquots (*ca.* 500 ng) were then digested (3 h, 37**°**C) with either RsaI or MspI (New England Biolabs). Preliminary tests were done with these soils evaluating fragment generation by RsaI, MspI, HhaI and AluI; RsaI and MspI generated the greatest number of fragments, and were selected for use in the full study. DNA Fragments were precipitated by addition of ice cold 100% ethanol and overnight incubation at −20**°**C. Analysis of digests was done with an Applied Biosystems 3730xl DNA analyzer (Applied Biosystems, Foster City, CA). A preliminary test was done to determine DNA mass loads that were within the optimal range of quantification for the instrument, and then each sample was analyzed in triplicate at the optimized mass loading. This procedure was adopted from preliminary tests examining variability at each stage of the TRF workflow, which showed that the fragment analysis was the most significant source of error. Fragments of <50 fluorescence units were removed from the analysis, and baseline noise were eliminated by an interactive procedure similar to that of Rees et al. [31] that first excluded fragments <1% of the total peak area, followed by re-normalization and elimination of fragments constituting <5% of the total peak area. The replicate profiles were aligned and TRF profiles of averaged fragment abundance were developed for each sample.

All analyses of TRF data were done with the multivariate statistical software package Primer v. 6 (Primer-E Ltd, Plymouth, UK). A Bray-Curtis coefficient was used to generate similarity matrices, which were ordinated by non-metric multi-dimensional scaling (MDS) computed with 100 random restarts. Analysis of similarity (ANOSIM) was applied to assess the magnitude and statistical significance of dissimilarity between soils in TRF profiles. The BEST (Bioenvironmental Step) routine was applied to identify high rank correlations between the TRF similarity matrices and a matrix of all edaphic factors determined in this study. The BEST analysis was configured to include up to six variables in generating correlations, and run 999 permutations for testing correlation significance.

### Construction of AOA and AOB clone libraries and bioinformatics

For all soils, aliquots of each of the replicate DNA extracts were pooled, and an aliquot of the pooled extract was used in PCR Amplification of archaeal *amo*A and bacterial *amoA* was done as described above for TRF analyses, except that the forward primers were not labelled. The PCR products were then purified using the QIAquick PCR Purification Kit (QIAGEN), cloned with the Zero Blunt TOPO system (Invitrogen), transformed into *E.coli* JM 109 competent cells (Promega, USA) and transformants were selected by plating on Luria Bertani medium containing ampicilin and X-gal (each at 100 µg mL^−1^). Sixteen clones were selected from each soil for sequencing. Plasmid templates in these clones were amplified by using the TempliPhi system (GE HealthcareLife Sciences, Pittsburgh, PA), and then inserts sequenced by using an M13 primer and the BigDye Terminator Cycle Sequencing system (Applied Biosystems). Reactions were analyzed with an Applied Biosystems 3730xl DNA analyzer.

Raw sequence files were imported into Geneious Pro 5.4 (Biomatters Ltd., Auckland 1010, New Zealand) for manual curation and translation. Libraries were examined for potential chimeric sequences with UCHIME (Edgar et al. 2011; http://www.drive5.com/uchime/) and sequence identities were determined by searching GenBank with the BLAST-N web server (http://www.ncbi.nlm.nih.gov/). Sequences were aligned with MUSCLE (Edgar 2004; http://www.drive5.com/uchime/). Phylogenetic trees were constructed by the neighbour-joining method and the Jukes-Cantor distance model, and were created with Geneious Pro v. 5.4.5. The bootstrap re-sampling method was used with 1000 replicates and minimum bootstrap values of 50. Phylogenetic trees were illustrated with FigTree (v. 1.3.1, http://tree.bio.ed.ac.uk/software/figtree/). Distance matrices were created with PHYLIP v. 3.69 (http://evolution.genetics.washington.edu/phylip.htm) and assignment of operational taxonomic units (OTU) and rarefaction analyses were done with Mothur (http://www.mothur.org, [32]). Principle coordinate analyses (PCoA) were done with Fast Unifrac *via* webserver (http://bmf.colorado.edu/fastunifrac/).

### Statistical analyses

Analysis of variance (ANOVA) was used to test for significant differences in nitrification potential between soils at the 95% confidence level with Genstat 13 (VSN International, Hemel Hempstead, HPI IESUK). Data from nitrification potential assays was examined for outliers by Grubbs' Test by using an online calculator (www.graphpad.com/quickcalcs/grubbs2). All other analyses were done by using Prism v. 5.4.5 (Graphpad Software, La Jolla, CA).

### Sequence accession numbers

Representative sequences of each archaeal *amoA* and bacterial *amoA* OTU are deposited in Genbank under accessions KF888663-KF888698 for archaeal *amoA* and KF888699-KF888724 for bacterial *amoA*).

## Results and Discussion

### Soil physico-chemical characteristics and nitrification potential

All soils were sampled from non-managed locations vegetated primarily by grasses *viz.* Elephant grass (*Pennisetum purpureum*) and Fowl Foot grass (*Eleusine indica*) as the predominant species. The exception was the Arena sandy loam which was sampled from a preserve of seasonal evergreen forest, which was originally predominant in Trinidad. The soils developed from a variety of different parent materials (Table S1 in File S1), ranged in texture from sandy loam to clay, spanned pH 4.8 to 8.2 and varied in a number of other physico-chemical characteristics (Tables 1, 2). Five acidic soils (pH<6) spanned textural classes, two circum-neutral soils (pH 6.2, 6.6) were loams, and two alkaline soils (pH 7.8, 8.2) were clays.

**Table 1:** Characteristics of soils used in this study^a^.

			Clay	Sand	Silt	TOC	TC	TN	TKN	NH_4_-N	NO_3_-N	BD	CEC
Soil Name	Texture	pH	------------------------------ g/kg ---------------------------------------							------- mg/kg ------		Mg/m^3^	cmol(+)/kg
Arena	Sandy Loam	5.4	4.57	74.00	21.43	6.1	8.90	0.65	0.7	16.4	2.1	1.78	0.3
Ecclesville	Silty Loam	4.8	21.81	34.2	43.99	26.2	3.91	3.46	3.2	46.9	56	1.54	8.8
Piarco	Silty Loam	4.8	17.71	32.00	50.28	13.6	17.87	1.56	1.5	20.5	6.65	1.58	3.1
Maracas	Loam	4.8	10.96	47.32	41.72	13.6	15.89	1.39	1.5	69.5	<0.01	1.56	0.9
River Estate	Loam	6.6	11.79	49.54	38.67	7.9	10.81	0.97	0.9	3.6	0.01	1.56	2.3
St. Augustine	Loam	6.2	19.76	39.16	41.08	24.5	24.75	1.58	1.5	16.4	35.6	1.52	5.6
Brasso	Clay	8.2	57.17	15.21	27.61	16.5	25.46	2.37	1.8	27.1	73.0	1.22	35.3
Princes Town	Clay	7.8	52.52	14.59	32.89	15.5	21.19	1.76	1.1	24.8	24.0	1.24	17.1
Talparo	Clay	5.8	46.71	17.32	35.97	21.3	29.81	2.69	2.3	52.0	46.1	1.28	29.1

a TOC, Total Organic Carbon; TC, Total Carbon; TN, Total Nitrogen; TKN, Total Kjeldahl Nitrogen; BD, Bulk Density; CEC, Cation Exchange Capacity.

**Table 2:** Soil Elemental Properties^a^.

Soil Name	P	K	Ca	Mg	S	Zn	B	Mn	Fe	Cu	Al	Na
	------------------g/kg-----------------------------					--------------------------------mg/kg-----------------------------------------------------------------------						
Arena	0.03	0.1	0	0.04	0.1	2.77	<2	4.31	1046.9	<0.5	642.9	17.6
Eccelville	1	2.8	5	3.5	0.4	112.2	<2	777.73	24453.3	32.13	22058.6	125.5
Piarco	0.9	1.2	2	0.3	0.2	41.45	<2	48.55	4724.8	14.35	12731.3	160.1
Maracas	0.5	0.6	0	0.5	0.2	23.87	<2	392.56	26648	34.76	11909.5	42.7
River Estate	0.3	0.8	1	0.9	0.1	28.23	<2	127.27	10617.5	11.1	6599.8	110.2
St.Augustine	1	1.9	2	0.4	0.2	126.3	<2	333.66	20695.8	34.49	12277.3	245.4
Brasso	0.9	4.5	10	5	0.5	130	5.48	604.38	34142.7	34.37	46458.4	198.6
Princes Town	0.5	4.1	15	8.2	0.3	165	8.2	977.84	31128.9	44.68	54124	204.5
Talparo	1.9	4.6	9	4.2	0.5	158.7	4.96	624.97	33908.2	28.45	45810.3	207.2

a Abbreviations: P = Phosphorus; K = Potassium; Ca = Calcium; Mg = Magnesium; S = Sulphur; Zn = Zinc, B = Boron;

Mn = Manganese; Fe = Iron, Cu = Copper; Al = Aluminium; Na = Sodium.

The three clay soils were vertisols, a defining characteristic of which is a high content of the swelling phylosilicate, montmorillonite. The remainder of the soils either had non-swelling kaolinite as the main phylosilicate mineral or contained relatively little clay (Table 1). Clay content and pH were significantly correlated (*p* = 0.0245) while pH and TC levels were not. Total carbon levels ranged from 8.90 g kg^−1^ soil to 39.13 g kg^−1^ soil, but TC was not correlated with clay content (*p* = 0.3317). The CEC was strongly correlated with clay content (*p* = 0.0003), but not levels of soil OC (*p* = 0.3438). Total N content and TC contents were strongly correlated (*p*<0.0001), but neither parameter was correlated with levels of either ammonium-N or nitrate-N.

Nitrification potential (NP) differed significantly among soils (ANOVA *p*<0.001, Table 3) with the Arena sandy loam being lowest at 0.6 mg N kg^−1^ soil d^−1^ and Brasso being highest at 14 mg N kg^−1^ soil d^−1^). Rates were linear over the incubation period with r^2^ = 0.952 to 0.998. Within the clays, there was a clear impact of pH on NP, as the rate in the pH 8.2 Brasso soil was nearly three times greater that in the pH 5.8 Talparo soil (Table 3). The high NP of the Brasso soil was a significant outlier (Grubbs Test *p*<0.01) from that of the other soils, and was thus excluded from the correlation analyses. The NP rates had a significant correlation (*p*<0.0001) with pH (Table 4) and with edaphic characteristics relating to soil texture/structure including: clay content, sand content, bulk density and cation exchange capacity (Table 4). The elemental composition also strongly correlated with NP, as only one element (P) of the 11 determined, lacked a signification relation to NP (Table 4). All of the carbon and nitrogen parameters measured were strongly correlated to NP, except total organic carbon and ammonium-N, which were not significant (Table 4). The significant positive correlation between NP and soil nitrate concentrations could indicate that variation in soil nitrate levels was at least in part attributable to varying rates of nitrification.

**Table 3:** Nitrification Potential^a^.

Soil	pH	mg N kg^−1^ soil d^−1^
Arena	5.4	0.7
Ecclesville	4.8	2.1
Piarco	4.8	0.6
Maracas	4.8	1.1
River Estate	6.6	2.4
St. Augustine	6.2	2.7
Brasso	8.2	14.1
Princes Town	7.8	6.6
Talparo	5.8	5.4

a N = NO_3_-N+NO_2_-N, *p*<0.001, LSD (5%) = 0.17, SEM = 0.06.

**Table 4:** Correlations between nitrification potential and edaphic properties^a^.

Property^b^	*p* value	r^2^
pH	<0.0001	0.5929
clay	<0.0001	0.8888
sand	<0.0001	0.5756
silt	0.3230	0.0444
TOC	0.1161	0.1085
Total C	0.0031	0.3342
Total N	0.0897	0.1253
TKN	0.6111	0.0119
NH_4_-N	0.7743	0.0038
NO_3_-N	0.0172	0.2319
BD	<0.0001	0.8612
CEC	<0.0001	0.7205
P	0.1897	0.0822
K	<0.0001	0.7887
Ca	<0.0001	0.8573
Mg	<0.0001	0.7965
S	<0.0019	0.3623
Zn	<0.0001	0.7404
Mn	<0.0001	0.5508
Fe	0.0001	0.4576
Cu	0.0012	0.3850
Al	<0.0001	0.8560
Na	0.0006	0.4206

a Correlations exclude the Brasso soil;

b See Table 1 for abbreviations.

The positive correlation of NP rates with soil pH observed in the present study data was consistent with the majority of the literature [33], [34], [35], [36]. But, our study also demonstrated that clay content had a significant factor modulating the pH effect. For example, the Arena sandy loam and Talparo clay were two acidic soils of roughly similar pH (5.4 and 5.8, respectively), yet NP in the montmorrillonic clay soil was *ca.* eight-fold greater than that of the sandy loam. Prior investigators determined that ammonia oxidation by AOB pure cultures was stimulated by expanding clays (montmorillonite) but not non-expanding clays, such as kaolinite [37], [38]. Jiang et al. [39] demonstrated that amending a kaolinitic Oxisol with montmorillonite greatly increased NP as well as abundance of both AOA and AOB. For both pure culture and soil studies, the stimulatory effect of montmorrillonite was hypothesized to reflect the ability of this high-CEC mineral to enable localized exchange of ammonium and protons and thereby buffer acidity [37]. Our study builds on prior findings from laboratory experiments, and illustrated with soil field samples the potential impact on nitrification of naturally existing levels of expanding clays like montmorillonite.

### Microbial community analysis by qPCR

**Figure 2:**
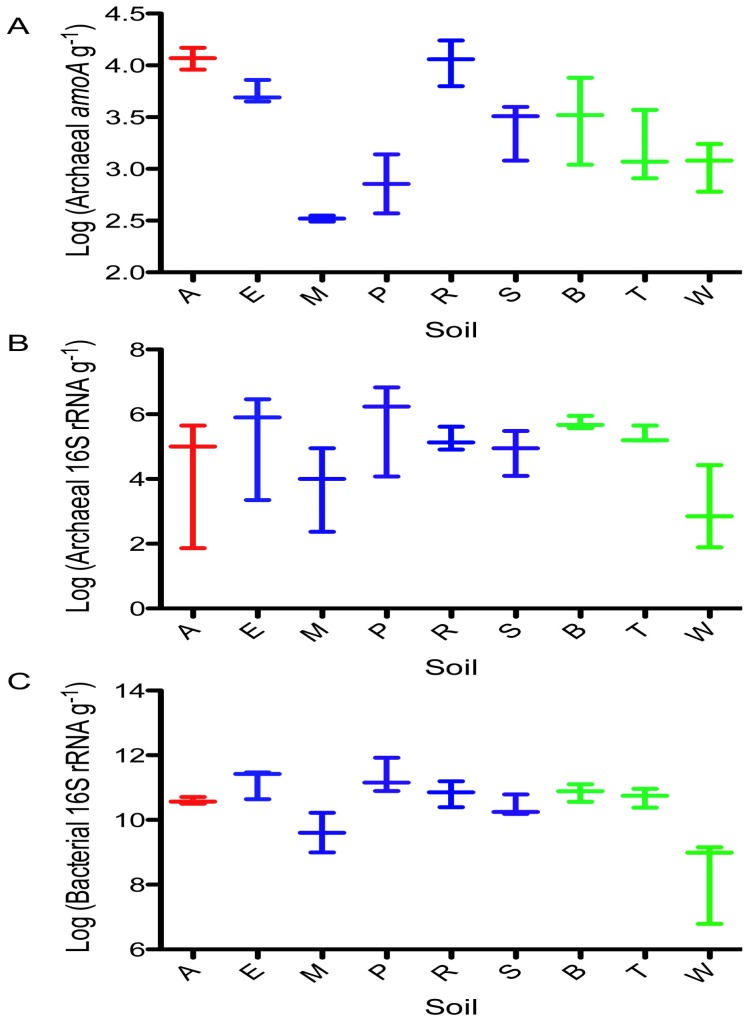
Box and whisker plots of gene abundance determined by qPCR. Panel A, Archaeal *amoA*; Panel B, Archaeal 16S rRNA; Panel C, Bacterial 16S rRNA. Symbol colors correspond to soil types: red, sandy loam; blue, silty loam; green, clay. Soil name abbreviations are: A, Arena; B, Brasso; E, Ecclesville; P, Piarco; R, River Estate; S, St. Augustine; T, Talparo; W, Princes Town.

The majority of the samples (93%) yielded amplification of archaeal *amoA*, which ranged in abundance from 300 copies (g soil)^−1^ in the Maracas loam to 11830 copies g^−1^ soil in the Arena sandy loam (Fig. 2A). The abundance of archaeal *amoA* showed significant negative correlations with: ammonium concentrations (r^2^ = 0.2504, *p* = 0.0079), OC levels (r^2^ = 0.2148, *p* = 0.0149) and clay content (r^2^ = 0.1589, *p* = 0.0394). Archaeal *amoA* abundance was also inversely related to total N and total carbon (*p* = 0.0569 and 0.0789, respectively). But, there was no significant correlation between archaeal *amoA* abundance and NP (*p* = 0.4371) or pH (*p* = 0.8794). In contrast to archaeal *amoA*, amplification of bacterial *amoA* occurred in only 33% of the samples (Table S2 in File S1). For most soils, amplification occurred in either just one of the three replicates or none of these (Table S2 in File S1). For example, the highest bacterial *amoA* abundance was 12,010 copies g^−1^ soil obtained from one of the three Piarco silty loam replicates, while the other two Piarco soil replicates did not yield bacterial *amoA* signals that were within the quantification limit (Table S2 in File S1). The low frequency of detection for bacterial *amoA* precluded assessing relationships between AOB abundance and soil properties. It should be noted that ammonia-oxidzers could exist that are not detected by currently available PCR primers.

Genes for bacterial 16S rRNA and archaeal 16S rRNA were amplified from all replicates of all soils. Archaeal 16S rRNA gene numbers ranged from 9.24×10^3^ copies g^−1^ soil in the Princes Town clay to 2.85×10^6^ copies g^−1^ soil in the Piarco silty loam (Fig. 2B). There were no significant correlations with archaeal 16S rRNA gene abundance and any soil properties. Bacterial 16S rRNA copies were *ca.* 10^6^-fold greater than archaea. Bacterial 16S rRNA gene numbers ranged from 8.1×10^8^ copies g^−1^ soil in the Princes Town clay to 3.5×10^11^ copies g^−1^ soil in the Piarco silty loam (Fig. 2C), the same two soils that showed minimum and maximum archaeal 16S rRNA gene abundance. There were no significant correlations with bacterial 16S rRNA gene abundance and any soil property, and there was no correlation between NP and the abundance of 16S rRNA genes for either archaea or bacteria.

The negative relation of AOA abundance with ammonium levels was in accordance with similar findings by prior investigations indicating a preference of soil AOA for low ammonium levels [18], [21], [40], [41], [42], [51]. Since all soils in the present study were from non-managed locations, the soil N dynamics were not impacted by N inputs derived from agricultural practices (*e.g*., mineral or organic N fertilizers, tillage, grazing). Therefore, differences in AOA abundance that arose from variation in ammonium levels resulted from N cycling processes intrinsic to the soils. Mineralization would be a major mechanism for ammonium input, and the present study thus supported the concept that a key factor favouring soil habitation by AOA *vs.* AOB is the predominance of mineralization as the N input pathway [12], [43].

The negative correlation of AOA abundance to OC levels could have reflected the preference of AOA for low organic nutrient conditions [44], [45]. But, soil organic matter affects a wide range of soil physico-chemical properties (*e.g.*, bulk density, water-holding capacity, aeration, *etc.*) that also influence microbial communities, and one or more of these broad soil properties cannot be ruled out as exerting effects on AOA. Similarly, clay minerals have many impacts on soil characteristics that affect microbial activity, which complicates interpretation of the negative correlation observed here of clay content with AOA abundance. In a survey of an organic farm, Wessen and colleagues [21] determined a negative correlation of AOA with clay content, which they theorized to reflect increased binding of ammonium, implying reduced ammonium bioavailability. But, the potential for clays to impede AOA growth *via* ammonium binding is unclear, as even ammonium tightly bound between interlayers of expanding clays (“fixed ammonium”) can be readily accessed by nitrifiers [46]. Also, as described above, expanding clays could have a stimulatory effect on nitrification. Thus, while a clay-associated inhibitory effect cannot be ruled out, we hypothesize that these minerals could exert a negative impact on AOA abundance by their effects on broader physico-chemical characteristics of soil (*e.g.*, bulk density, water-holding capacity, aeration, *etc.*).

The AOB populations in these tropical soils were low, and at levels not readily assayed by qPCR. Similarly low AOB populations have been reported by other investigators [7], [43], [47]. For example, Pett-Ridge and co-workers [47] were unable to amplify bacterial *amoA* from soils of a tropical forest and, in a survey of soils across Scotland, AOB were below qPCR detection limits in *ca.* 62% of the 184 sites sampled [17]. Thus, for soils having AOB populations below qPCR detection, AOA have been inferred to be the primary group driving nitrification [7], [43], [47]. By this reasoning, AOA are implicated as dominant drivers of nitrification in the tropical soils studied here. While AOA appeared to play a key role in nitrification, AOA numbers were not correlated with NP. A lack of correspondence between nitrification levels and nitrifier community density (AOB and/or AOA) has been reported by many other investigators [23], [43], [47], and the reasons for this disconnection are unknown. But, as mentioned above, it's possible that ammonia-oxidzers exist that are divergent from known betaproteobacterial AOB and thaumarchaeal AOA, and thus evade detection by PCR primers developed from currently available sequence data.

### Analysis of community composition by *amoA* sequencing and TRF

**Figure 3:**
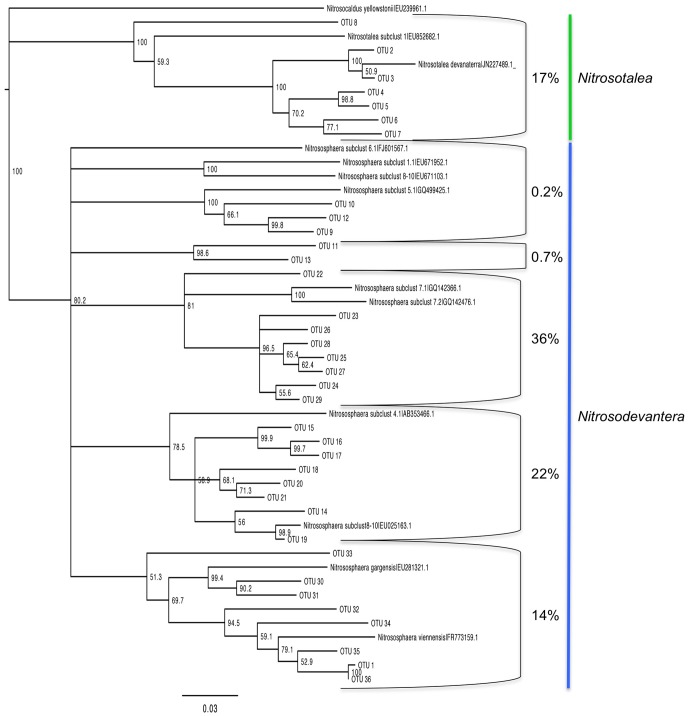
Neighbour joining consensus tree of archaeal *amoA* phylotypes. Colored branches indicate the *Nitrosotalea* cluster (green) and *Nitrososphaera* cluster (blue). Boot strap values are indicated at nodes. Cluster designations follow the classification of Pester et al. [25] based on nucleic acid alignment of archaeal *amoA*.

**Figure 4:**
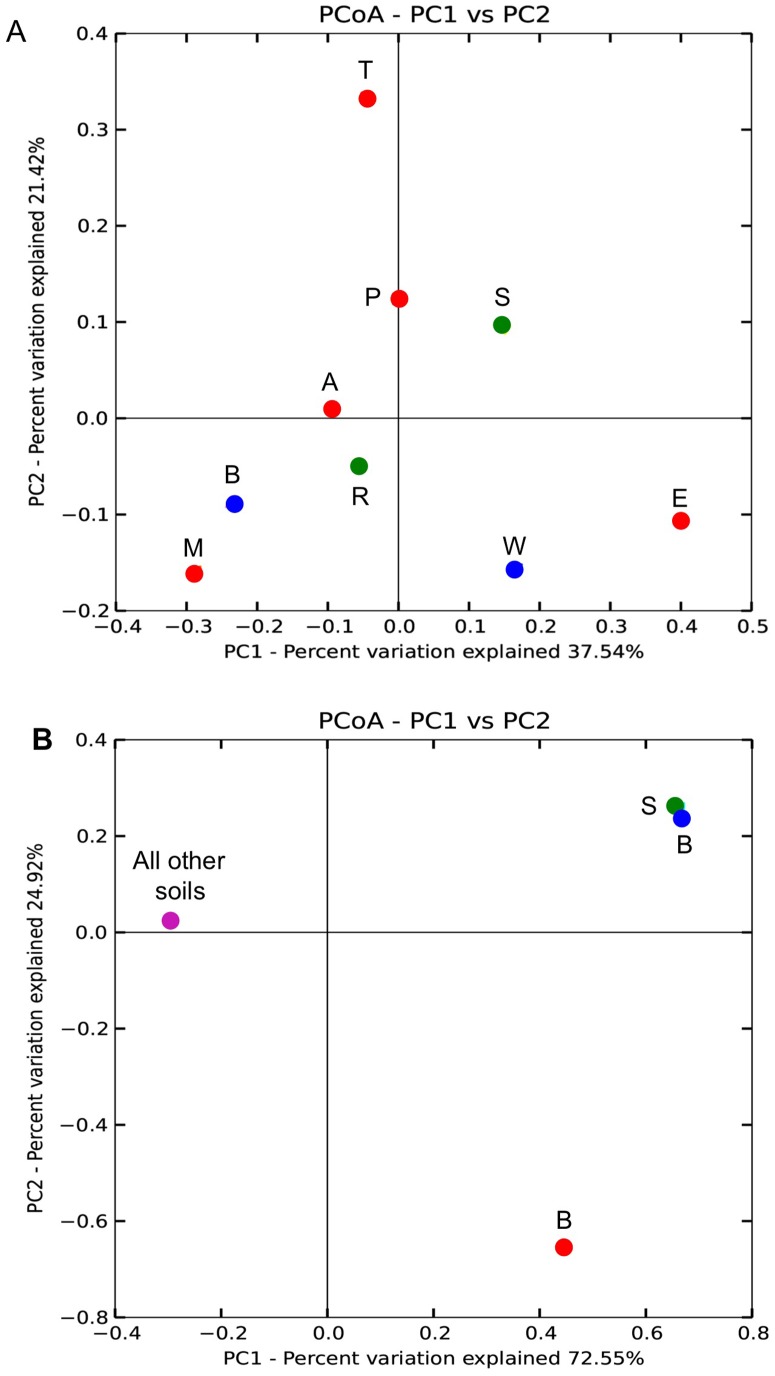
Principle coordinate plot of archaeal *amoA* clone libraries (Panel A) and bacterial *amoA* clone libraries (Panel B). Symbol colors correspond to soil pH: red, acidic (pH<6); green, circum-neutral (pH 6.2, 6.6), blue, alkaline (pH 7.8, 8.2), purple, multiple pH. Letters indicate soil names that are abbreviated as: A, Arena; B, Brasso; E, Ecclesville; P, Piarco; R, River Estate; S, St. Augustine; T, Talparo; W, Princes Town.

Sequencing of archaeal *amoA* clone libraries generated 35 OTUs from a total of 121 sequences (Table S3 in File S1, Fig. S1). Most phylotypes (83%) were identified as *Nitrososphaera* and the remainder as *Nitrosotalea*. The largest group of sequences (36%) were associated with *Nitrososphaera* subclusters 7.1 and 7.2 (Fig. 3; Table S3 in File S1), and identified in all soils except the Princes Town clay (Table S3 in File S1). Sequences grouping wih *Nitrososphaera* subcluster 4 and *Nitrososphaera* subclusters 8–10 ranked second in abundance accounting for 22% of the sequences (Fig. 3; Table S3 in File S1). The *Nitrosotalea* phylotypes were identified in clone libraries from the St. Augustine, Maracas and River Estate loams, but were most abundant in the latter two (Table S3 in File S1). Analysis of the archaeal *amoA* phylotypes by PCoA showed no significant correlation with pH (Fig. 4A). Furthermore, while all *Nitrosotalea* phylotypes were identified from loams, there was no consistent soil characteristic that explained their more frequent detection in these soils.

**Figure 5:**
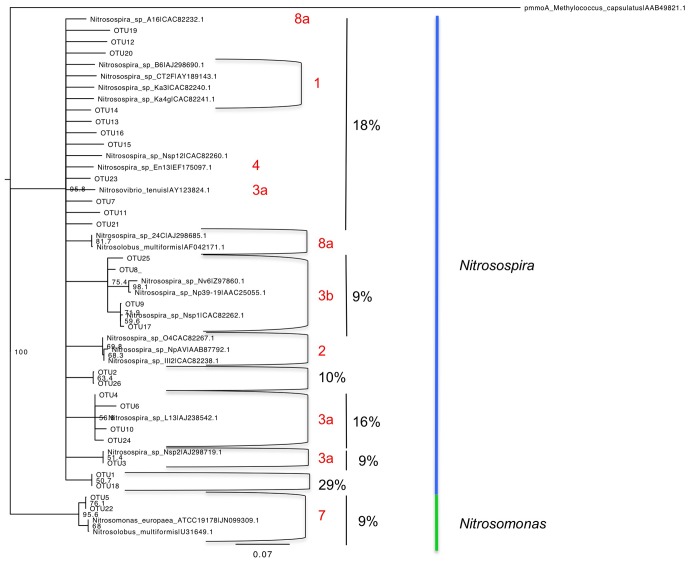
Neighbor joining consensus tree of bacterial AmoA phylotypes. Colored branches indicate the *Nitrosomonas* cluster (green) and *Nitrosospira* cluster (blue). Boot strap values are indicated at nodes. Values in red are cluster designations following the classification of Avrahami et al. [48] based on amino acid alignment of bacterial AmoA.

Sequencing of bacterial *amoA* clone libraries generated 26 OTUs from a total of 128 sequences (Table S4 in File S1). Most OTU (91%) were *Nitrosospira* phylotypes with the remainder associated with the *Nitrosomonas* (Fig. 5). Of the *Nitrosospira* phylotypes, 27% were affiliated with Cluster 3a and Cluster 3b (Fig. 5), and the remainder did not group within AOB clusters defined by Avrahami and colleagues [48]. Among those not grouping with a defined cluster was the largest phylotype (OTU 1), which comprised 29% of the sequences and occurred in all soils except the St. Augustine loam and Talparo clay (Table S3 in File S1). Analysis of bacterial *amoA* phylotypes by PCoA showed no significant corrrelation of phylotypes with pH (Fig. 4B). While one alkaline soil and one cirum-neutral soil clustered apart from one acidic soil, the rest of the soils, representing a range of pH, did not segregate (Fig. 4B).

**Figure 6:**
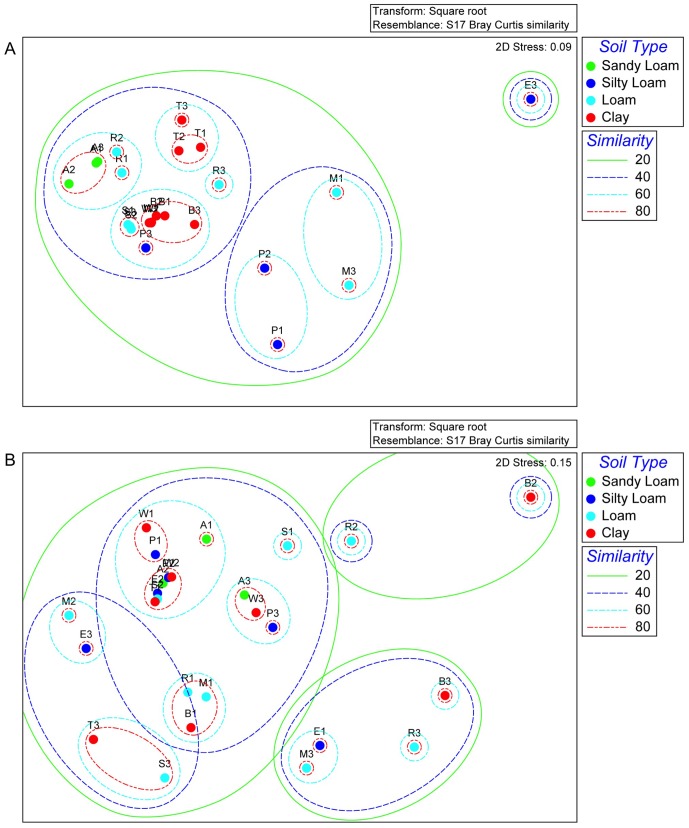
Non-metric multidimensional scaling plots of archaeal *amoA* terminal restriction fragment profiles generated by digestion with MspI (Panel A) or RsaI (Panel B). Symbol colors correspond to soil types, letters indicate soil names and values are the replicate number for the indicated soil. Soil name abbreviations: A, Arena; B, Brasso; E, Ecclesville; P, Piarco; R, River Estate; S, St. Augustine; T, Talparo; W, Princes Town.

In TRF analysis of archaeal *amoA*, RsaI profiles were more effective than those of MspI in differentiating soils by MDS (Figs. 6A,B). The ANOSIM global R of the RsaI profiles was 0.752 (*p*<0.001) indicating significant dissimilarity between soils, whereas for the MspI profiles the ANOSIM global R was 0.058 and not significant. Comparison of the observed TRF phylotypes with those predicted from archaeal *amoA* sequences indicated that the majority of TRF mapped to the above-mentioned *Nitrososphaera* phylotypes. One *Nitrosotalea*-affiliated TRF (RsaI 315) was most abundant in the River Estate soil, which also had the highest proportion of *Nitrosotalea*-like archaeal *amoA* clones. For archaeal *amoA*, BEST analysis identified the combination of ammonium levels and total Kjeldahl N (TKN) as most important in explaining variation in the RsaI profiles (correlation 0.630, *p*<0.001, Table 5). Nitrogen-related variables were a dominant factor, as ammonium appeared in all ten of the significant combinations, and was accompanied by TKN and/or TN in all but one. Four other factors (Zn, TC, pH, P) were included in some explanations with these N variables, but none yielded a more significant correlation with TRF similarity patterns than did the combination of ammonium with either TKN or TN (Table 5).

**Table 5:** BEST variables explaining variation between soils in archaeal *amoA* RsaI terminal restriction fragment profiles^a^.

No. Variables	Correlation	Variable						
		NH_4_	TN	TKN	Zn	TC	pH	P
2	0.630	X		X				
2	0.624	X	X					
3	0.620	X		X	X			
2	0.619	X				X		
3	0.618	X	X		X			
3	0.618	X	X					
3	0.613	X	X				X	
5	0.612	X	X			X	X	X
4	0.611	X	X	X	X			
4	0.609	X		X		X	X	

a Variables included in correlation indicated by “X”. Abbreviations: P, Phosphorus; Zn, Zinc; TC, Total Carbon; TN, Total Nitrogen; TKN, Total Kjeldahl Nitrogen.

**Figure 7:**
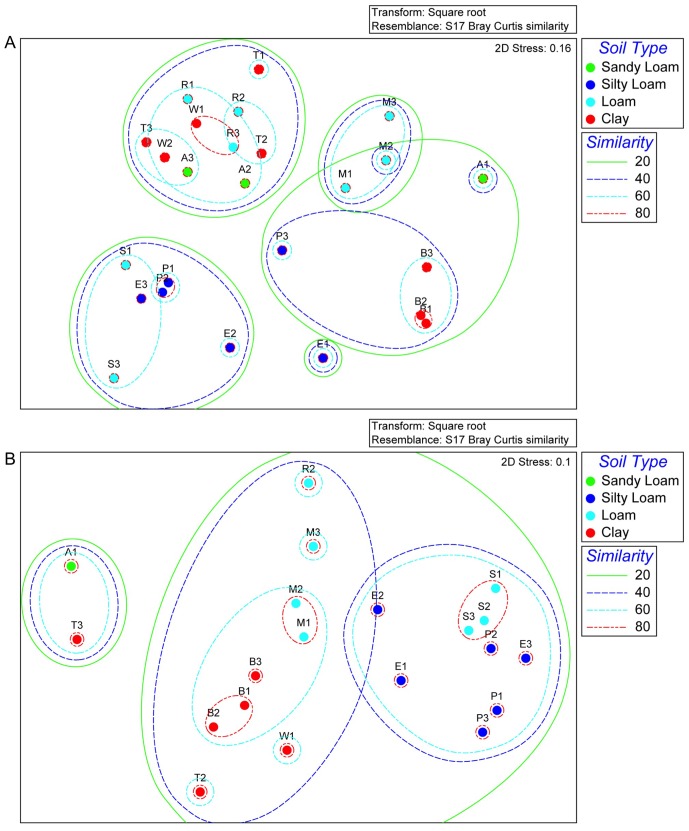
Non-metric multidimensional scaling plots of bacterial *amoA* terminal restriction fragment profiles generated by digestion with MspI (Panel A) or RsaI (Panel B). Symbol colors correspond to soil types, letters indicate soil names and values are the replicate number from the indicated soil. Soil name abbreviations: A, Arena; B, Brasso; E, Ecclesville; P, Piarco; R, River Estate; S, St. Augustine; T, Talparo; W, Princes Town.

In the TRF analysis of bacterial *amoA*, soil differentiation was more effective with MspI profiles than with those of RsaI (Figs. 7A,B). The former had an ANOSIM global R of 0.819 (*p*<0.001) indicating significant dissimilarity between groups. But, the RsaI ANOSIM Global R of 0.058 was not significant. From BEST analysis of the bacterial *amoA* MspI profiles, TRF phylotype variation across the soils was best explained by phosphorus (P), bulk density (BD) and Fe (correlation 0.648, *p*<0.001, Table 6). These variables were dominant factors explaining MspI TRF profile variation, as P occurred in all ten correlations, BD in nine and Fe in eight (Table 4). Aluminium was also prominent, and was included in five correlations (Table 6). Other significant factors were Cu, Na, clay, S, B, OC, and K. Notably, neither pH nor any N-related variable was included in a significant correlation.

**Table 6:** BEST Variables explaining variation between soils in bacterial *amoA* MspI terminal restriction fragment profiles^a^.

No. Variables	Correlation	Variable^a^										
		P	BD	Fe	Al	Cu	Na	Clay	S	B	OC	K
3	0.648	X	X	X								
3	0.632	X		X	X							
4	0.623	X	X	X	X							
2	0.621	X	X									
6	0.619	X	X	X			X	X	X			
5	0.618	X	X	X	X	X						
5	0.617	X	X	X		X				X		
4	0.616	X	X		X	X						
5	0.612	X	X	X	X						X	
6	0.609	X	X	X			X	X				X

a Variables included in correlation indicated by “X”. Abbreviations: P = Phosphorus; K = Potassium; S = Sulphur; B = Boron; Mn = Manganese; Fe = Iron, Cu = Copper; Al = Aluminium; Na, Sodium; BD, Bulk Density; OC, Organic Carbon.

Our analysis of archaeal *amoA* clone libraries was consistent with prior reports establishing *Nitrososphaera* as a cosmopolitan soil inhabitant [5], [7], [49]. But, our identification of *Nitrosotalea* phylotypes in soils of widely varying pH (and other edpahic factors) contrasted with prior studies, which have indicated *Nitrosotalea* as associated primarily with acidic soils [25]. Furthermore, PCoA analyses of archaeal *amoA* clone libraries did not support the hypothesis that pH was the primary factor in AOA phylotype selection, and contrasted with results from prior investigators [3], [7], [50]. For bacterial *amoA* clone libraries, predomination by *Nitrsosospira* was congruent with the findings of other investigators [23], [48], [49]. But, as with AOA, the data did not confirm the hypothesized effect of pH as a major driver of AOB community structure.

Collectively, results from the BEST and qPCR analyses indicated that communities of AOA and AOB were shaped by differing sets of edaphic factors. The study conducted by Wessen et al. [52] revealed a similar conclusion, although specific soil characteristics that differentially affected these communities were not identified. In the present study, soil N characteristics (ammonium-N and organic N pools) were the major factor affecting AOA communities, but were not identified to affect those of AOB. This finding was significant as it provided field-based evidence that AOA ecophysiology was responsive to natural variation in N levels, data which is limited in the current literature. Also, absence of soil N as a factor affecting AOB would be consistent with adaptation of AOB to relatively high N inputs, which were lacking in the soils studied here. If so, our field-based data would support the concept that soil N levels are one factor important in affecting niche separation of AOA and AOB.

For AOB, the dominant edaphic factors affecting community structure were the combination of P, BD and Fe. Soil P level was the only variable identified to affect communities of both AOA and AOB, but it was much more significant for the AOB. Phosphorus levels have been previously identified as affecting AOB communities [19], [20], [24]. But, those effects emerged in response to P enrichment through exogenous P application. In contrast, in the present study, the effect of P was associated with variation in P levels endogenous to the soils. The eco-physiological significance of the P effect on AOB community structure is unclear. It may reflect a direct impact on some aspect of AOB physiology and/or indirect effects, such as competition for P with other organisms [19], [20], [24]. The effect of BD on AOB could have reflected one or more impacts of BD on soil characteristics, perhaps most importantly oxygen diffusion. The strong effect of iron on AOB community structure might have reflected the iron-heme dependent respiration of AOB [53], [54], and possibly indicated iron acquisition was a limiting factor. The BEST analyses identified Cu and Al as community structure determinants for AOB. Both metals could have impacted community structure *via* toxicity effects, but the significance of Cu could also have reflected its core role in AOB biology as a component of ammonia mono-oxygenase and other enzymes in N metabolism [53], [54]. However, since Cu and Al were intercorrelated with Fe levels, it's difficult to resolve metal-specific effects.

## Conclusions

Detectable *amoA* genes of AOA predominated over detectable bacterial *amoA* genes in these tropical soils, and their community structure was affected primarily by soil N characteristics. In contrast, communities of the low abundance AOB were shaped by edaphic factors other than soil N. Contrary to our hypothesis, pH was not a major factor affecting abundance or structure of either AOA or AOB communities. Nitrification levels were strongly affected by soil pH and clay content, but not by the abundance of AOA or AOB, or aspects of AOA or AOB community structure. Thus, edaphic factors other than pH differentially affected evolution and niche differentiation of AOA and AOB communities. But, the impact of pH (and clay content) on nitrification levels appeared to more strongly reflect physico-chemical effects on AOA or AOB activity, rather than alterations in ammonia-oxidizer abundance or selection for AOA or AOB phylotypes differing in nitrifying capacity.

## Supporting Information

Figure S1 **Rarefaction curves for clone libraries of archaeal** ***amoA*** **(Panel A) and bacterial** ***amoA*** **(Panel B).**
(TIFF)

File S1 **Supporting tables.** **Table S1.** Soil Taxonomy and Soil Sites Sampled. **Table S2.** Amplification of Bacterial *amoA* by qPCR. **Table S3.** Distribution of archaeal *amoA* OTU by soil. **Table S4.** Distribution of bacterial *amoA* OTU by soil.
(XLSX)
