# Supplementary figures and images for: Impacts of Edaphic Factors on Communities of Ammonia-Oxidizing Archaea, Ammonia-Oxidizing Bacteria and Nitrification in Tropical Soils
Source: PLoS One. 2014 Feb 28;9(2):e89568. doi: 10.1371/journal.pone.0089568 (PMC3938500; doi:10.1371/journal.pone.0089568)

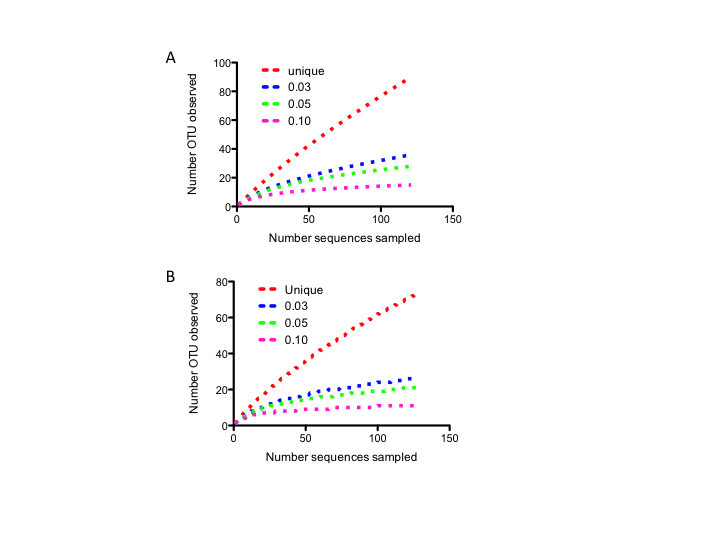

Supplement: Figure S1 — Rarefaction curves for clone libraries of archaeal amoA (Panel A) and bacterial amoA (Panel B). (TIFF) [file pone.0089568.s001.tif]
